# Patient Perspectives of Quality of the Same-Day Antiretroviral Therapy Initiation Process in Gauteng Province, South Africa: Qualitative Dominant Mixed-Methods Analysis of the SLATE II Trial

**DOI:** 10.1007/s40271-020-00437-4

**Published:** 2020-09-10

**Authors:** Nancy A. Scott, Mhairi Maskew, Rachel M. Fong, Ingrid E. Olson, Alana T. Brennan, Matthew P. Fox, Lungisile Vezi, Peter D. Ehrenkranz, Sydney Rosen

**Affiliations:** 1 Department of Global Health, Boston University School of Public Health, 801 Massachusetts Ave 3rd Floor, Boston, MA 02118 USA; 2 Health Economics and Epidemiology Research Office, Department of Internal Medicine, School of Clinical Medicine, Faculty of Health Sciences, University of the Witwatersrand, Johannesburg, South Africa; 3 Department of Epidemiology, Boston University School of Public Health, Boston, MA USA; 4 Bill & Melinda Gates Foundation, Seattle, WA USA

## Abstract

**Background:**

HIV patients in South Africa continue to report operational barriers to starting antiretroviral therapy (ART). In the Simplified Algorithm for Treatment Eligibility (SLATE) II trial, same-day initiation (SDI) of ART increased the number of patients commencing ART and achieving HIV viral suppression by using a screening tool to distinguish between patients eligible for SDI and those requiring additional care before starting treatment. We conducted a mixed-methods evaluation to explore trial patients’ perceptions and experiences of SDI.

**Methods:**

SLATE II was implemented at three urban, public primary health care clinics in Gauteng Province, South Africa. We conducted a short quantitative survey and in-depth interviews among a purposive sample of 89 of the 593 trial participants in the intervention and standard arms, using a mixed inductive–deductive framework approach.

**Results:**

Nearly all respondents (95%) were satisfied with their care, despite reporting clinic wait times of ≥ 3 h (72%). Intervention patients found the initiation process to be easy; standard patients found it complicated and were frustrated with being shuffled around the clinic. No intervention arm patients felt that SDI was “too fast” or indicated a preference for a more gradual process. Both groups highlighted the need for good counselling and non-judgmental, respectful staff. Standard patients suggested improving patient–provider relations, strengthening counselling, reducing wait times, and minimising referrals.

**Conclusions:**

While it is difficult to untangle the role of providers from that of the SLATE algorithm in influencing patient experiences, adoption of SLATE II implementation procedures could improve patient experience of treatment initiation.

**Trial registration:**

Clinicaltrials.gov NCT03315013, registered October 19, 2017.

**Electronic supplementary material:**

The online version of this article (10.1007/s40271-020-00437-4) contains supplementary material, which is available to authorized users.

## Key Points for Decision Makers

**Table:** 

Standard procedures for initiating antiretroviral therapy (ART) for treatment of HIV in South Africa can be confusing to patients, who must make multiple clinic visits and are often shuffled around a clinic to receive different services.
Same-day ART initiation using a structured process such as that evaluated in this study is not “too fast” for most patients.
Regardless of the speed of the initiation process, patients prefer non-judgmental, respectful clinic staff, high-quality counselling, privacy during the clinic visit, and fewer referrals within the clinic.

## Background

Both the World Health Organization (WHO) [1] and the South African National Department of Health [2] recommend same-day initiation (SDI) of antiretroviral therapy (ART) for people living with HIV considered to be eligible and ready, with “same-day” typically referring to the day of HIV diagnosis or the first clinic visit thereafter, if diagnosed outside a facility. Offering SDI of ART has been shown to increase the number of patients commencing ART and increase the number of patients achieving HIV viral suppression [3–7].

Although ART initiation procedures have been simplified in recent years [8], patients in South Africa still report experiencing operational barriers to starting treatment, such as long wait times, multiple pre-initiation visits, staff shortages, and poor communication between providers and patients [9–11]. The Simplified Algorithm for Treatment Eligibility (SLATE I and II) trials addressed some of these operational barriers using a screening algorithm intended to safely and rapidly identify patients eligible for SDI, while correctly identifying those who required additional care before starting ART [3, 12, 13]. Results from the SLATE I trial, conducted in Kenya and South Africa, suggested the algorithm could improve rapid ART treatment initiation, but was too conservative; many patients were unnecessarily identified for further screening before ART initiation [3]. Building upon SLATE I, the SLATE II algorithm was refined to better differentiate between patients eligible for SDI and those requiring further care prior to ART initiation [13]. The SLATE II study was a non-blinded, individually randomised trial assessing a clinical algorithm designed to increase and accelerate the uptake of treatment and improve outcomes among adult (≥ 18 years), non-pregnant, HIV-positive patients presenting at public-sector clinics in South Africa. Among the patients randomised to the intervention arm, a proportion was eligible to start ART immediately (SDI), at the same clinic visit, per the algorithm. Other patients randomised to the intervention arm were referred for further standard of care services before ART initiation because the algorithm identified symptoms, conditions, or other reasons for additional investigation. Patients randomised to the standard arm received standard of care at the study clinics [13].

To explore patients’ perceptions of operational barriers and facilitators in the ART initiation process and of SDI using the SLATE II algorithm in South Africa, we conducted a qualitative dominant mixed-methods study [14] on a subset of SLATE II patients during passive follow-up.

## Methods

### Study Setting and Participants

SLATE II was implemented at three urban, high-volume, public primary health care clinics in Gauteng Province of South Africa. Protocols and results for the SLATE I and SLATE II studies can be found elsewhere [3, 12, 13, 15]. Between December 2018 and April 2019, we conducted a cross-sectional survey using qualitative dominant mixed methods [14] among a purposive sample of SLATE II study participants in both study arms at all three study sites. We administered a brief quantitative survey and conducted semi-structured in-depth interviews (IDIs) to existing SLATE II patients who presented for a routine clinic visit during the follow-up data collection period. Patients were approached by study staff and invited to participate until we enrolled our target sample size. As prior studies indicated that a sample size of at least 12–15 IDIs is recommended to ensure sufficient saturation or predictability of qualitative interview responses [16, 17], we aimed to conduct 15 IDIs per study arm per site, for a total of 90 participants. Among the intervention group at each site, we aimed to interview 12 who were eligible for SDI and three who were not, roughly proportionate to overall SLATE II enrolment patterns.

### Data Collection and Management

Six multilingual study staff trained in qualitative interviewing methods and human participant research ethics conducted the interviews in each patient’s preferred language. Following a set of quantitative closed-ended questions about the perception of service quality measured on a five-point Likert scale, qualitative open-ended, non-leading questions were used to prompt further discussion on three key themes: (1) quality and acceptability of ART initiation processes; (2) facilitators and barriers to ART initiation and adherence; and (3) suggestions to improve the ART initiation process. Interviews lasted approximately 20–45 min and were audio-recorded with consent from the patient, translated (if necessary) and transcribed verbatim into a password-protected Microsoft^®^ Word document. Audio recording was not required, but all patients consented to being audio-recorded and interviewers took supplemental field notes as needed to understand any important non-audible context. The quantitative survey was captured on paper, then entered into tablets using RedCAP Mobile [18].

### Conceptual Framework

To understand what patients perceive as the most salient elements of quality, we used an adapted version of the conceptual framework proposed by the WHO, “Quality of the service experience” [19]. This framework was used to assess patients’ satisfaction and their perspectives on key domains of quality of the ART initiation process and their early treatment experience. The framework is comprised of three main sections: (1) programme effort, (2) elements of quality, and (3) impacts. We limited this analysis to the six ‘elements of quality’ (*choice*, *information given*, *technical competence*, *interpersonal relations*, *mechanisms to encourage continuity*, and *appropriate constellation of services*) and applied these to the patient experience in the SLATE II trial (Table 1).

**Table 1:** WHO adapted ‘elements of quality’ domains as applied to the SLATE II intervention. Adapted from: World Health Organization. Quality of care in the provision of sexual and reproductive health services: evidence from a WHO research initiative [Internet]. Geneva, Switzerland; 2011. Available from: www.who.int/reproductivehealth

Elements of quality	Applied definitions of the conceptual framework for the SLATE II intervention
1. Choice (availability and variability)	1. Availability and variability of HIV services and medication at service-delivery points
2. Information given to clients	2. Patients receive counselling before ART initiation to ensure that patients understand essential information such as what it means to be HIV positive, how to prevent spread of HIV, treatment instructions, and the importance of adherence
3. Technical competence	3. Providers are trained and are able to apply current HIV clinical practices (recommended clinical practices and evidence-based practices)
4. Interpersonal relations	4. Provider–patient relationship and interaction; provider attitude while with patient
5. Mechanisms to encourage continuity	5. Providing linkages to other related HIV services and referrals; integration of HIV services with other related services
6. Appropriate constellation of services	6. Organisation of HIV services is convenient and acceptable to the patients, including costs

*ART* antiretroviral therapy, *SLATE* Simplified Algorithm for Treatment Eligibility, *WHO* World Health Organization

### Data Analysis

Quantitative data were analysed using SAS software v.9.3 (SAS Institute Inc., Cary, NC, USA). We describe respondent descriptive characteristics from the main SLATE II database and report summary statistics for closed-ended survey questions, stratified by study arm. Patient satisfaction scores were reported for the friendliness of staff, privacy during visits, overall understanding of ART initiation, and overall satisfaction with care.

Qualitative analysis was managed in NVivo 12 (QSR International, Doncaster, Australia). The Framework Method was used to code and analyse the qualitative data using a mixed inductive–deductive approach [20]. First, an initial codebook was created; codes were identified a priori according to the three key themes in the interview guide and the conceptual framework domains. Second, two researchers (RMF, IEO) familiarised themselves with the interviews and then independently coded the transcripts line-by-line to the themes and framework domains. Additional codes were identified during the coding process as new themes emerged from the data. After discussion and agreement was reached, codes with similar content were merged. The final codebook can be found in Electronic Supplementary Material 1.

The coded data were charted using the matrix query in NVivo 12 (QSR International, Doncaster, Australia) against the quality of care framework to identify patterns of ideas and concepts related to the key quality domains. The two researchers (RMF, IEO) then summarised and interpreted the patterns and selected salient quotations to support the findings. Results were first discussed with the first author (NAS) and then with other authors. The qualitative data were then triangulated with the quantitative results from the satisfaction survey. Deviations from the common themes and patterns were captured and analysed further to investigate explanations for atypical responses. To mitigate researcher bias, the two coders coded a selection of the same transcripts. Similar codes were merged and discrepancies were discussed and resolved, and findings were reviewed by the field team.

This analysis focuses on the structural and implementation-related clinic-level barriers that the SLATE II intervention aimed to address. The qualitative findings from the two study arms (standard of care arm, intervention arm stratified by those eligible for SDI and those not) are compared and presented by the key themes: patient perceptions on the quality and acceptability of the ART initiation process, facilitators of and barriers to ART initiation and adherence, and suggestions for improvement. Results are reported using the Standards for Reporting Qualitative Research (SRQR) checklist [21].

## Results

### Respondent Demographics

We conducted 89 of the targeted 90 IDIs. Two interviews were omitted from the analysis due to audio recording problems, leaving an analytic sample of 87. Of these, 43 patients were drawn from the standard population, 37 from intervention arm patients eligible for SDI, and seven from intervention arm patients not eligible. Table 2 presents the demographic characteristics of respondents.

**Table 2:** Participant characteristics from the SLATE II qualitative sub-study

Characteristic	Standard arm (*n* = 43)		Intervention arm eligible for same-day initiation (*n* = 37)		Intervention arm not eligible for same-day initiation (*n* = 7)		Total (*N* = 87)	
Age in years (median, IQR)	36 (32–43)		36 (29–42)		39 (31–45)		36 (31–43)	
Sex (female), *n* %	26	60.5	25	67.6	3	42.9	54	62.1
Marital status, *n* %								
Single	24	55.8	21	56.8	3	42.9	48	55.2
Married or long-term partner	15	34.9	14	37.8	4	57.1	33	37.9
Divorced or widowed	4	9.3	2	5.4	0	0	6	6.9
Location patient currently resides, *n* %								
Informal urban	40	93.0	37	100	7	100	84	96.6
Urban	3	7.0	0	0	0	0	3	3.4
Number of other persons in the household (median, IQR)	1 (1–2)		1 (1–3)		1 (0–2)		1 (1–2)	
Employment status, *n* %								
Employed (formal)	10	23.3	8	21.6	4	57.1	22	25.3
Employed (informal)	11	25.6	5	13.5	2	28.6	18	20.7
Unemployed, looking for work	19	44.2	20	54.1	1	14.3	40	46.0
Other	3	7.0	4	10.8	0	0	7	8.0
Study site, *n* %								
Site A	13	30.2	13	35.1	2	28.6	28	32.2
Site B	15	34.9	12	32.4	2	28.6	29	33.3
Site C	15	34.9	12	32.4	3	42.9	30	34.5

*IQR* interquartile range, *SLATE* Simplified Algorithm for Treatment Eligibility

### Patient Satisfaction

**Fig. 1:**
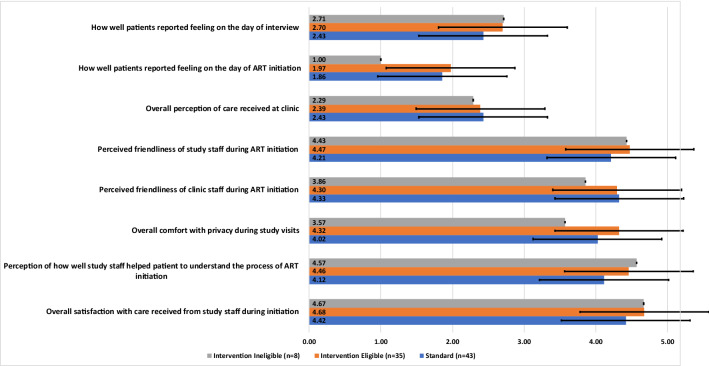
Patient satisfaction responses at time of interview (mean, SD). *ART* antiretroviral therapy, *SD* standard deviation

We observed few differences in perceptions of key quality and satisfaction domains reported on Likert scales between the groups (Fig. 1). The SLATE II algorithm determined eligibility for SDI largely on the basis of a patient’s clinical condition on the day of study enrolment. Patients with severe symptoms of tuberculosis or other illnesses, for example, were not eligible for SDI. Not unexpectedly, therefore, all intervention patients who were not eligible for SDI reported feeling worse on the day of study enrolment compared to the other groups (Fig. 1).

Almost all respondents were satisfied or very satisfied with the care they received from study staff on the day they began the ART initiation process (95%), perceived the study staff to be friendly or very friendly (80%), and felt comfortable or very comfortable with the amount of privacy during their study visits (88%) (data not shown). Almost all respondents also perceived the clinic staff to be friendly (90%), despite reporting waiting three or more hours on average for any given clinic visit (72%). About 90% of patients believed that they had a good or very good understanding of the ART initiation process after their interaction with study or clinic staff.

### Qualitative Results

All 87 interviews in the analytic sample were coded. Code saturation [22], which was determined when no new codes were identified after several interviews, was likely reached slightly before all interviews were coded.

In response to qualitative questions about things that make it easy for people in their communities to initiate or adhere to ART early in treatment, patients in all groups identified individual-level facilitators (e.g. acceptance of HIV status, encouraged/motivated to live a healthy life), interpersonal-level facilitators (e.g. family and peer support, openness with partner), and community-level facilitators (e.g. encouragement from church, reduced stigma, community is accepting). Patients in all groups also identified similar barriers to initiation and early treatment adherence, primarily around knowledge and attitudes (e.g. lack of knowledge about HIV and taking ART, unwillingness to change lifestyle, fear of side effects) and structural factors (e.g. food insecurity, community stigma). However, qualitative responses differed between groups, with regard to the elements of quality at the clinic level (Table 3). Illustrative quotes support the key themes that emerged from the patient IDIs (Table 4), organised by the element of quality and study arm.

**Table 3:** Perceptions of quality and acceptability of ART initiation process by patient type from SLATE II

Quality element	Standard arm	Intervention arm eligible for same-day initiation	Intervention arm not eligible for same-day initiation
Choice			
Positive	Most perceived choice of when to start treatment: patients are given option of whether to initiate treatment the same day or wait for CD4 results	Patients are asked about readiness to start treatment Study staff stressed not forcing patients to do anything they do not want to	No themes emerged
Negative	Few perceived not having a choice for same-day initiation	No themes emerged	No themes emerged
Information			
Positive	Patients perceived counselling as acceptable: received information about HIV, test results, medication, side effects, and lifestyle changes such as stopping smoking and drinking, eating well Counselling helped patients accept their status	Found counselling helpful to understand medication time, side effects, no CD4 count threshold, using condoms Counselling helped patients overcome fear and gain comfort and courage Felt encouraged to ask questions	Learned same-day initiation was a possibility Counselled about starting/taking ART
Negative	Perceived inadequate counselling Confusion around same-day initiation	Lacked understanding of tests	
Patient perception of provider’s technical competence			
Positive	Generally met patient expectations Patients thought counsellors were very good and well trained	Met patient expectations, got correct tests, medication Patients felt better treatment by the SLATE study staff than the clinic staff Patients surprised and satisfied they could initiate ART the same day Patient grateful to receive a shopping voucher	Still report receiving medication promptly (medication for other illness) High quality of care
Negative	Few had expectations of same-day initiation; thought providers were wasting time by not initiating them on treatment Some patients reported lost test results, lost files, incorrect information, wrong medication	Felt poor quality of service at the clinic (not being checked by the doctor, not assisted, lost file, asked to come back later)	
Interpersonal relations			
Positive	Generally positive interaction with counsellors and nurses; friendly, professional, comfortable, supportive, treated patient well, non-judgmental	Study staff were welcoming and comforting: allowed patients to be open and feel respected, did not feel rushed Liked the privacy of study staff not being from their own community	Treated well, staff caring and patient
Negative	Some reported negative interaction with providers; provider did not show any concern or cannot speak to patients properly	Some expressed concern that clinic staff will be rude/treat them disrespectfully because of their status	No themes emerged
Mechanisms to encourage continuity			
Positive	Believed patients were more likely to initiate and continue taking ART if perceived good service at clinic Referred to hospital or followed up at clinic for TB care and other health problems Referred to pharmacy for medication pick up	Perceived that same-day initiation facilitates ART initiation Patients liked not having to be referred Requesting pills for three months at a time	Patients referred to the clinic side for treatment of various illnesses (TB, shingles, headache)
Negative	Believed patients were less likely to continue if long queue/wait time Patients sent from one place to another at clinic—too many places; few were not able to initiate same day	None	None
Appropriate constellation of services			
Positive	Perceived process as acceptable Same-day initiation unexpected Medication pick up is easier, more options for pick up location; it was fast, did not take long—used to long wait Given specific time to pick up medication	Same-day treatment is easier for people who work or are unmotivated to come back to start treatment; SLATE treatment was faster than the clinic is usually	Pleased with short wait time, felt process was easy
Negative	No privacy when collecting medication Cards and files are different colours for HIV patients at the clinic	Clinic is full and less organised, patients feel they wait too long in the queues, sometimes do not receive medication and have to come back Cards are different colours for HIV patients at the clinic	Long wait, even just to receive treatment at the clinic

*ART* antiretroviral therapy, *SLATE* Simplified Algorithm for Treatment Eligibility, *TB* tuberculosis

**Table 4:** Illustrative quotes on the perceptions of quality and acceptability of the ART initiation process

Quality element	Study arm	Quote
Information	Intervention arm eligible for same-day initiation	(a) “The counselling was the best part, because I was scared to start on the medication, but the counselling made me strong.”
Information	Intervention arm not eligible for same-day initiation	(b) “She [SLATE II staff] encouraged me so much that I really asked many questions when I came in the first time. After that I was also able to encourage my wife to get tested and a cousin of mine to speak to me about her health.”
Information	Standard arm	(c) “I was given the results. She [the counsellor] then counseled me and explained what the numbers meant. She also explained to eat this and do this and that. She told me I would have to stop smoking and drinking.”
Technical competence	Standard arm	(d) “I did blood tests three times and they [clinic staff] told me that the blood samples have gone but mine was late, that’s the issue I had. Even last year, I did blood tests but I never got the results. I even went there and they checked on the computer…she kept writing things down, looking on the computer.”
Interpersonal relations	Standard arm	(e) “When you get there, they [clinic staff] give you the number of the room where you would be given medication. You don’t feel comfortable because you don’t even know what is going on. They [providers] show no concern. I wish they could put nurses who deal specifically with patients who are coming in to take treatment for the first time.”
Interpersonal relations	Standard arm	(f) “They [the nurses] were very friendly. They asked if I had tested before and I was honest to say that I have not. They told me it is important to know my status so I could get treatment. They were honest and friendly. They have love.”
Interpersonal relations	Intervention arm not eligible for same-day initiation	(g) “When you get there they [SLATE staff] talk to you, they ask you questions, they are free. They don’t harass you. If you want to tell them something you can open up.”
Interpersonal relations	Intervention arm eligible for same-day initiation	(h) “They [SLATE staff] have good communication skills and they know how to treat a patient unlike the nurses at the clinic, who shout at people.”
Mechanisms to encourage continuity	Intervention arm eligible for same-day initiation	(i) “I think it [SLATE II process] is acceptable for the community. You test and you get the results…there is nothing else you can do but start the pills. What would make a person not go back to the clinic is if they come back after a month. People change their minds and don’t go back and stay at home.”
Appropriate constellation of services	Standard arm	(j) “I didn’t find it easy, it was difficult. You don’t get help from one place, you have to go to different places. When you get somewhere, they send you somewhere else.”
Appropriate constellation of services	Intervention arm eligible for same-day initiation	(k) “From what I experienced with SLATE, I realised that if SLATE II [staff] were the ones working full time, people would not be complaining like they do at the clinic, because you would even get excited to go get treatment.”
Appropriate constellation of services	Intervention arm eligible for same-day initiation	(l) “There is a huge difference. The normal clinics are very full and you have to wait for a very long time. Some people cut the queue; some are fighting. Here [SLATE II office], it is a small place and you get attended to immediately.”
Appropriate constellation of services	Intervention arm eligible for same-day initiation	(m) “Everything you do is confidential. They [SLATE II staff] don’t shout ‘people here for ART come to this side’. So I would like for people with HIV to experience the service we get here.”

*ART* antiretroviral therapy, *SLATE* Simplified Algorithm for Treatment Eligibility

#### Elements of Quality

Patients from the standard, intervention same-day eligible and intervention same-day ineligible groups generally spoke positively about the elements of quality during their ART initiation process and early in treatment (Table 3).

Intervention patients perceived they had a *choice* about initiating treatment and felt study staff focused on patient readiness during the process. Standard responses did not converge; most standard group patients felt they were given an option while a few perceived they were not.

In terms of *information given to clients,* the intervention groups found counselling helpful in providing them with information on ART, side effects, and lifestyle changes, but also felt it helped patients overcome fear and gain comfort, something not reported by the standard patients (Table 4; quotes a and b). The intervention ineligible patients learned that, despite being referred for further care, SDI was a possibility. On the other hand, standard group patients described counselling as being given all the necessary information they needed about their status and how to take their medication, but suggested that clinic providers could take more time to answer questions and ask patients how they are doing (Table 4; quote c).

Patients in all groups generally found the *technical competence* of the clinic staff to be acceptable, though intervention patients spoke more positively about the quality of service provided than did the standard group. Several patients in the standard group perceived the clinic staff to be incompetent, citing lost test results or patient files, delayed treatment initiation, and, in one instance, being given the wrong ART medication (Table 4; quote d).

In reference to *interpersonal relations,* defined as the provider–patient interaction and provider attitude while with the patients, almost all patients across all groups described SLATE II study staff as welcoming, comforting, patient, and non-judgmental and that they treated patients well, which allows patients to be open and feel respected (Table 4; quotes g and h). While the standard group patients generally reported positive interactions with clinic staff during the initiation process, many felt staff did not show any concern and “cannot speak to patients properly” (Table 4; quotes e and f). Some intervention arm patients not eligible for SDI expressed a similar concern about the clinic staff.

More themes emerged around *mechanisms to encourage continuity,* or the linkages to other related HIV services, from the standard group than from either of the intervention groups. The standard group reported patients were more likely to initiate and continue care if they perceived good service, if the clinic had shorter lines, and if there were fewer referrals to other services within the clinic. Patients in the intervention groups reported not having to be referred as an important facilitator to continuity of care. More importantly, intervention eligible patients perceived SDI to facilitate ART initiation, with one patient giving the example that once a patient leaves the clinic, they may change their minds and not return to the clinic for treatment (Table 4; quote i).

One key difference between intervention and standard patients pertains to the *appropriate constellation of services,* or the organisation of services as perceived by the patient. Standard group patients perceived the initiation process as complicated, noting long wait times at the clinic and being constantly shuffled around to receive different services, which one patient described as, “this is how you lose patients” (Table 4; quote j).

Some standard group patients were pleased with the process, having not expected SDI, and expressed that they were accustomed to the typically long wait times at the clinic. On the other hand, intervention eligible patients discussed the benefits of not being referred and getting all services at the same place, which makes the visits shorter than is typical at the clinic (Table 4; quotes k and l). While quantitative results show no significant difference in wait times across study arms, respondents qualitatively perceived differences.

Corroborating the quantitative findings, standard group patients also expressed concern over a lack of privacy during their clinic visit, reporting that patient files were colour-coded and they had stood in a particular line, making them vulnerable to being identified as HIV positive by other patients. Patients in the intervention arm acknowledged the same concerns, but did not report experiencing this during their visit (Table 4; quote m). Rather, they reported liking that study staff were not from their own surrounding communities.

#### Suggestions for Improvement

When asked for suggestions to improve the ART initiation process and early adherence experience, patients in all groups offered actionable suggestions (Table 5). Regarding the initiation process, while the Likert scale indicated high satisfaction, in the interviews, standard group patients heavily discussed improving patient–clinic relations, improving counselling, and reducing wait times. Specifically, they recommended having a nurse who is dedicated to handling new patients in order to improve the quality of the counselling and provider–patient relations. Additionally, they suggested minimising referrals within the clinic in order to not confuse and frustrate patients. Intervention eligible patients echoed these suggestions and also recommended community sensitisation efforts focused on generating demand for testing and increasing awareness around SDI availability. Both standard group patients and intervention eligible patients also suggested all patients be tested for HIV regardless of visit type and that all positive patients receive SDI (Table 5).

**Table 5:** Patients’ suggestions to improve ART initiation process and early adherence, stratified by patient type

Themes	Standard arm	Intervention arm eligible for same-day initiation	Intervention arm not eligible for same-day initiation
Suggestions to improve ART initiation process	Improve counselling and testing options Have a separate nurse who can have focused time with new patients Make home testing available Improve processes at clinic Simplify the experience (fewer referrals) so that they do not lose patience, get confused Do not colour-code files Cut down on wait times, hire more staff Same-day treatment after testing positive Community sensitisation efforts Educate youth and community on HIV and the benefits testing and treatment	Improve counselling and testing options Test all patients for HIV regardless of visit type Ensure everyone has SLATE II counselling experience Mobile clinics Improve processes at clinic Do not isolate HIV patients at clinic; not standing in two lines Do not colour-code files Same-day treatment for everyone Community sensitisation efforts Door-to-door education campaign Tents for counselling and testing near schools, churches, taverns Address HIV in church, political parties Advertise ART on TV	No themes emerged
Suggestions to improve early adherence	Improve privacy and process at clinic Do not have separate lines for different illnesses Do not colour-code files Make it easier for returning patients to see doctor if any problems Improve counselling and follow-up options Home visits Check in via call or SMS Improve drug collection options Be able to receive ART by post Address food insecurity Government to provide food parcels for unemployed	Improve counselling and follow-up options Staff the clinic to reduce wait times Follow-up with patients (phone call, SMS reminders, or home visits) Patients should set an alarm/reminder On-going counselling at the clinic Better explain tests and patient file before seeing the doctor Improve drug collection options Make drug collection possible at community outlets Make separate place in the clinic for collection only	Improve privacy and process at clinic Increase number of staff Improve counselling and follow-up options Establish support groups Home visits Clinic should call if a patient does not pick up medication Improve drug collection options Collect medication from machines Would like medication for more than one month at a time Address food insecurity Provide food vouchers Government should create more jobs

*ART* antiretroviral therapy, *SLATE* Simplified Algorithm for Treatment Eligibility

In response to improving early adherence, there were similar patient responses across all three groups. Patients discussed strategies to improve privacy and processes at the clinic and offered various suggestions to improve counselling and early follow-up and ideas to make drug collection easier on the patient. The standard and intervention ineligible groups offered suggestions to address food insecurity, including food vouchers and job creation (Table 5).

## Discussion

South Africa adopted Universal Testing and Treatment over 2 years ago, but the WHO guidelines offered little in terms of a specific implementation strategy for countries to efficiently and safely determine eligibility [1]. The SLATE II trial tested a simple screening algorithm that allowed a large proportion of patients to start treatment on the same day, while still effectively identifying those who required additional care first. Primary trial outcomes, however, focused on patients’ clinical success in initiating ART and remaining in care, without reference to how patients experienced the treatment initiation process.

This study using mixed data collection methods conducted among a sample of SLATE II participants found that patients’ perceptions of SDI and treatment were positive and consistent with opinions found in previous studies [10, 11, 23]. They generally perceived the ART initiation process and early treatment experience to be acceptable, while citing common individual- and social-level barriers to seeking care [23]. The main differences between intervention and standard group patients emerged around structural barriers at the clinic, including perceptions of patient–provider relations, quality of counselling, lack of privacy, confusing initiation procedures, and long wait times.

The importance of the patient–provider relationship in HIV treatment services is well documented in the qualitative literature both in South Africa and in the region more broadly [10, 11]. A patient who has just tested HIV positive will be heavily influenced by her or his immediate experience with the provider, as this point of contact is the first intersection of several quality domains, including *choice, information, interpersonal relations,* and *appropriate constellation of services*. Respondents suggested that while receiving information on medication, side effects, and lifestyle modifications is critical, feeling respected, comfortable to ask questions, and empowered to choose when and how to initiate treatment improves their motivation. Intervention group patients more frequently discussed high-quality counselling and emotional support that helps patients accept their status, ready them for initiation, and encourage adherence.

Perceived operational barriers to initiating treatment, those related to *mechanisms to encourage continuity* and *appropriate constellation of services*, were minimal among the intervention groups. The opportunity to receive all health services in one place, in private, and not having to wait all day made the ART initiation process easier. However, a lack of privacy, confusing initiation procedures, administrative mistakes, and long wait times were perceived as challenges experienced at the clinic by all patients, aligned with the literature more broadly [11, 23]. The algorithms used in SLATE I and SLATE II were designed to make treatment initiation simpler for both providers and patients and, if adopted for routine care use, could address some of the facility-specific barriers to initiation. Designating a nurse or a team of clinic staff who is trained intensively for interactions with first-time patients may also address concerns about the quality of counselling and patient–provider interactions.

Patient-generated solutions to address early adherence included community-based pick-up points, a quick line to collect medication at clinics, machines dispensing medication, and the ability to collect more than 1 month’s supply. Many of these strategies are already being implemented to varying degrees as outlined under the decentralised medical delivery policy [24] and the National Chronic Disease Adherence Guidelines [25], though they are not currently available to patients newly initiated on treatment. One additional benefit of enrolling more patients in these decentralised medical delivery interventions is that it may provide the opportunity for staff to spend more time with patients who need it, such as those newly initiating or those who are sick. Patients suggest that improved counselling, easier access to clinic staff, and intensified early follow-up efforts during early treatment could improve early retention, before patients can collect multiple months of medication or transition to the community drug collection strategies.

### Limitations

There are several limitations to this study. First, data were collected toward the end of SLATE II study follow-up, leaving room for recall bias by asking patients to discuss their treatment initiation experience anywhere from 3 to 12 months earlier. Second, data on quality perceptions were only collected from the patient perspective at one point in time, limiting our ability to understand the change in perceptions over time or to triangulate with provider perspectives. Third, as we recruited patients for the interviews from those attending clinic visits, the results reflect the experiences of patients who have returned to the clinic and have been retained on treatment after the study visit, and do not capture the perceptions and experiences of patients who did not return to the clinic and were lost from care after initiating ART. Those lost from care may have reported more negative experiences and have different suggestions for improving initiation and early adherence. Even though the interviews were conducted in a private and safe space, there was a risk of respondents not wanting to share information that they considered confidential or potentially harmful. Fourth, the purposive sampling for the qualitative interviews and the location of the overall SLATE II trial in only three primary health care clinics in one province limits the external validity of the findings. Lastly, researcher bias is possible, but it was somewhat mitigated as the interviewers were not the same research staff as those who analysed the data, the results were reviewed and discussed amongst authors, and the SRQR checklist was followed to report our research.

Despite the limitations mentioned above, our findings highlight several important points. First, patients offered SDI do want it; they appreciate that their time is not being wasted and that the process is clearer to them than it is with current standard care. Standard clinic procedures, such as being referred from service to service within the same clinic and waiting in multiple queues, cause frustration and discouragement. Based on the responses to our questions, the importance of clinic staff, including clinicians and counsellors, being perceived as friendly, approachable, and caring cannot be overstated.

## Conclusion

Based on our research, it is difficult to untangle the role of respectful, compassionate service providers from that of the SLATE algorithm itself in generating positive patient experiences. Study nurses and counsellors were trained and supervised to provide high-quality care, as per the study protocol, and did not face many of the pressures placed on clinic staff. At the same time, none of the specific tasks required for initiating ART under the SLATE II algorithm are different from those expected of clinic staff, and there is little that prevents clinic staff assuming more respectful, supportive behaviour. Both better procedures (the SLATE II algorithm) and better attitudes are needed, and future research should focus on generating both.

## Electronic supplementary material

Below is the link to the electronic supplementary material.

Supplementary material 1 (PDF 1136 kb)

Supplementary material 2 (DOCX 28 kb)

## Abbreviations

ART: Antiretroviral therapy
IDI: In-depth interview
SDI: Same-day initiation
SLATE: Simplified Algorithm for Treatment Eligibility
WHO: World Health Organization

## Declarations

### Availability of data and materials

The datasets used and/or analysed during the current study are available from the corresponding author on reasonable request.
