# Supplementary material for: Patient Perspectives of Quality of the Same-Day Antiretroviral Therapy Initiation Process in Gauteng Province, South Africa: Qualitative Dominant Mixed-Methods Analysis of the SLATE II Trial
Source: Patient. 2020 Sep 10;14(2):175–86. doi: 10.1007/s40271-020-00437-4 (PMC7884580; doi:10.1007/s40271-020-00437-4)
Supplement: Supplementary file 1 — Supplementary material 1 (PDF 1136 kb) [file 40271_2020_437_MOESM1_ESM.pdf]

SLATE II UID

Instrument ID: ID_1

Improved simplified clinical algorithm for identifying patients eligible for immediate initiation of antiretroviral therapy for HIV (SLATE II) – qualitative component

Patient In-Depth Interview Guide

Target Audiences: IDIs to be conducted among the following at all three SLATE sites

1) Patients who have consented to and are currently participating in SLATE II (intervention or standard arm) and have consented to the qualitative sub-study

Instructions for the Interviewer

Step 1: Introduction:
Interviewer: *Greet the patient. Briefly introduce yourself and the study in the patient's language of choice.*
"Thank you for agreeing to speak with me about potentially participating in an interview. As a reminder, we are discussing your experience with ART initiation including what you found useful and what you think might be improved. Would you like to proceed with the informed consent process?"

Does the patient wish to proceed?

YES ________ **(Proceed with step 2, informed consent process)**

NO ________ **(STOP! Thank the participant for their time. Do NOT proceed with informed consent)**

Step 2: Informed consent:

Interviewer: *Begin the informed consent process as per the training. If consent is granted, leave a copy of the information sheet with the participant. Confirm that the study ID is on each consent form and retain the signed consent and audio consent forms.*

Was informed consent obtained and documented in writing or a thumb print (including agreement for audio recording)?

YES ________ **(Proceed with step 3, introduction and interview)**

NO ________ **(STOP! Thank the participant for their time. Do NOT proceed with the interview)**

Step 3:

Please fill in the details below and begin the interview. When you have finished all relevant sections of the interview please double check that all the required questions have been answered. Thank the participant for the participation and time and complete the time that the interview finished below. As you ask the questions, please probe appropriately to gain as much depth on the topics as possible.

1. **Interviewer Initials** __________
2. **Interview date (DD/MM/YYYY)** __________
3. **Time start** __________
4. **Supervisor initials** __________
5. **Re-confirm participant study ID**__________
6. Study Arm (confirm with study staff): __(in) ___ (out)_____ Intervention arm; _______Standard arm

SLATE II UID

***STEP 4: Interviewer**: Start the audio recorder. Read the following statement. Please repeat the statement translated into the local language based on primary languages.*

"This is the audio-recording for participant **[SAY BARCODE UID NUMBER OUT LOUD]** being conducted on **[DAY, MONTH, YEAR**]. Thank you for agreeing to participate in this interview. My name is ____________. I will be asking you the questions about your experience with ART initiation. We will discuss what you found useful, what you think might be improved in the future, and your views on barriers or facilitators to the ART initiation process. Please feel free to tell me whatever you are comfortable sharing. You should also remember that you do not have to share anything that you are not comfortable sharing. We will not write down your name, only the study ID that you have from SLATE II. There are no right or wrong answers, so please be honest and tell me what is true for you. Are you ready to begin?"

***Interviewer:** "I'm going to start by asking you some basic questions about your ART initiation. In these questions, I will tell you whether I am asking about your experience with the study team (SLATE nurse and assistants) or with the clinic staff. Please try to tell me exactly who you are describing when you answer (clinic nurse, study nurse, etc.).*

Think back to when you first started the ART initiation process. Do you remember that day at the clinic? Okay, let's begin... "

Q#	Question	Code	Skip
1	How well do you feel today?	1= Very poor (very bad)<br>2= Poor (bad)<br>3= Ok<br>4= Good<br>5= Very good	
2	How well did you feel on the day of ART initiation?	1= Very poor (very bad)<br>2= Poor (bad)<br>3= Ok<br>4= Good<br>5= Very good	
3	On average how long do you wait in the clinic queue/waiting room for your appointment(s) for all the services you receive in a visit?	1= Less than one hour<br>2= 1-2 hours<br>3= 3 hours or more	
4	Overall how would you rate the care that you receive at this facility? (Note that this is about the clinic, not the study.)	1= Very poor (very bad)<br>2= Poor (bad)<br>3= Ok<br>4= Good<br>5= Very good	
5	When did you first test for HIV?	Month/year	
6	Where did you first test for HIV?	1= At this facility<br>2= At other facility in district<br>3= At other facility in province<br>4= Mobile clinic<br>5= At other facility in SA<br>6= Outside of SA<br>7= Campaign testing<br>8= Home testing<br>9= Other (specify)	
7	Have you started treatment for HIV (ART) yet?	0=No<br>1=Yes	If no, skip to question 11
8	If yes, how many months ago did you start?	Number of months	

9	When you enrolled in the SLATE II study, how many times had you already come to this clinic for HIV-related care, including having an HIV test or any other services?	1 = Day I enrolled in the study was the first time<br>2 = Day I enrolled in the study was the second time<br>3 = Day I enrolled in the study was the third time<br>4 = Day I enrolled in the study was the fourth time<br>5 = Day I enrolled in the study was the fifth time<br>6 = I have been to the clinic more than five times before enrolling in SLATE II	
10	Are you a participant in the SLATE II intervention arm or standard arm?<br>*Interviewer: confirm response is consistent with "Step 3, question 6". If inconsistent, resolve with study staff before proceeding.*	1 = Intervention arm<br>2 = Standard arm	If 1, proceed to question 11. If 2, skip to question 17.
**Intervention Participants only:**			
11	In addition to the study staff (our study assistants and nurse), which clinic staff did you see at the time of initiating HIV treatment? (Tick all that apply)	1= Clinic nurse (not study staff)<br>2= Clinic counsellor<br>3= Doctor<br>4= Pharmacist or PA<br>5= Other (specify)	
12	How friendly/welcoming/respectful were the study staff that you saw at the time of initiating ARV treatment?	1= Very poor (very bad)<br>2= Poor (bad)<br>3= Ok<br>4= Good<br>5= Very good	
13	How friendly/welcoming/respectful were the health care professionals that you saw at the time of initiating ARV treatment?	1= Very poor (very bad)<br>2= Poor (bad)<br>3= Ok<br>4= Good<br>5= Very good	
14	After being enrolled in SLATE, overall, how comfortable were you with the privacy you've had during the study visits?<br>*Interviewer: note this does not include consenting and enrolment into study*	1= Extremely comfortable<br>2= Not comfortable<br>3= Neither comfortable nor uncomfortable<br>4= Comfortable<br>5= Very comfortable	
15	Overall, how well did the study staff help you understand the process of ART initiation?	1= Very poor (very bad)<br>2= Poor (bad)<br>3= Ok<br>4= Good<br>5= Very good	
16	Overall how satisfied were you with the care you received from the study staff on day began the process of initiating ART?	1= Extremely dissatisfied<br>2= Not satisfied<br>3= Neither satisfied nor dissatisfied<br>4= Satisfied<br>5= Very satisfied	
**Standard arm participants only:**			
17	In addition to the study staff (our study assistants and nurse) you saw for enrolling into SLATE II, which clinic staff did you see at the time of initiating ARV treatment? (Tick all that apply)	1= Clinic Nurse (not study staff)<br>2= Lay counsellor<br>3= Doctor<br>4= Pharmacist or PA<br>5= Other (specify)	

18	How friendly/welcoming/respectful were the study staff that you saw at the time of initiating ARV treatment?	1= Very poor (very bad)<br>2= Poor (bad)<br>3= Ok<br>4= Good<br>5= Very good	
19	How friendly/welcoming/respectful were the health care professionals that you saw at the time of initiating ARV treatment?	1= Very poor (very bad)<br>2= Poor (bad)<br>3= Ok<br>4= Good<br>5= Very good	
20	After being enrolled in SLATE II, when you went to the clinic, overall how comfortable were you with the privacy you've had during your visits?<br>*Interviewer: note this does not include consenting and enrolment into study.*	1= Extremely comfortable<br>2= Not comfortable<br>3= Neither comfortable nor uncomfortable<br>4= Comfortable<br>5= Very comfortable	
21	Overall, how well did your clinic health care provider (counselor, nurse, or other clinic staff) help you understand the process of ART initiation?	1= Very poor (very bad)<br>2= Poor (bad)<br>3= Ok<br>4= Good<br>5= Very good	
22	Overall how satisfied were you with the care you received from the clinic staff on day began the process of initiating ART?	1= Extremely dissatisfied<br>2= Not satisfied<br>3= Neither satisfied nor dissatisfied<br>4= Satisfied<br>5= Very satisfied	

Theme 1: Quality and acceptability of the ART initiation process

Remind the participant that their input is very valuable and EMPHASISE ON THEM BEING EXPECTS

1.1 Pretend you're back at the day that you enrolled in SLATE II. Can you describe for me, as best you remember, the details of what happened after you consented to be in the study?

Probes for intervention arm	Probes for standard care arm
1. Providers they saw (study staff, clinic staff)<br>2. General experience: process, wait time, steps<br>3. Algorithm components: Symptom report, medical history, physical exam, readiness assessment, TB<br>4. Referred for further care before starting ART (screened out)? If yes, go to standard arm probes.	1. Providers they saw (clinic staff)<br>2. Experience at the clinic: process, wait time, steps<br>3. Services provided –medical history, physical exam, counseling, TB symptom screen ,other?<br>**4.** Referred for additional care? If so, what?

1.2 Did process to start ART meet your expectations and needs? Why or why not?

Probes for intervention arm	Probes for standard care arm
1. Did you (or will you) start ART when you thought you would? Why or why not?<br>2. How did you find the quality of the services you received on the day you started the process of ART initiation? What was the best part? Why? What was the worst part? Why?<br>3. Did you find the study/ SLATE process easy or complicated? What specifically made it easy? What things specifically made it complicated?	1. Did you (or will you) start ART when you thought you would? Why or why not?<br>2. How did you find the quality of the services you received on the day you started the process of ART initiation? What was the best part? Why? What was the worst part? Why?<br>3. Did you find the clinic process easy or complicated? What specifically made it easy? What things specifically made it

(*Interviewer: remind them of the process they described above)*	complicated? (*Interviewer: remind them of the process they described above)*

1.3 Would you hope that other people in your community who are HIV positive but have not yet started ART have the same experience as you? Please explain.

Probes for intervention arm	Probes for standard care arm
1. Is the SLATE II algorithm doing the right thing to support ART initiation in your community? Why or why not?<br>2. What would you change about your experience to make it better for someone else?<br>3. Is the SLATE II algorithm an acceptable approach for people in your community? Why or why not?	1. Are the health facilities doing the right thing to support ART initiation in your community? Why or why not?<br>2. What would you change about your experience to make it better for someone else?<br>3. Is the standard of care an acceptable approach for people in your community? Why or why not?

Theme 2: Barriers and facilitators to initiation and adherence

1.4 What are some of the things that make it easy for people in your community to initiate ART treatment? To adhere? What are some of the things that make it difficult to initiate? To adhere? (*Interviewer: List them, then probe for time, cost, support, drug collection options, adherence options*)

Easy	Challenges
Initiate:<br><br><br><br>Adhere:	Initiate:<br><br><br><br>Adhere:

1.5 Tell me a story about something specific that you felt made it easy for you to initiate treatment? To adhere to your medications?

Probes for intervention arm	Probes for standard care arm
1. Understanding the process through counseling, job aids, etc<br>2. Time, cost, efficiency of the SLATE II process<br>3. Support of family, friends or support groups<br>4. Drug collection options, support groups (adherence question)	1. Understanding the process through counseling, job aids, etc<br>2. Time, cost, efficiency of the standard care<br>3. Support of family, friends or support groups<br>4. Drug collection options, support groups (adherence question)

1.6 Tell me a story about something specific that you felt made it difficult for you to initiate treatment? To adhere to your medications?

Probe for:

Probes for intervention arm	Probes for standard care arm
5. Understanding the process through counseling, job aids, etc<br>6. Time, cost, efficiency of the SLATE II process<br>7. Support of family, friends or support groups<br>8. Drug collection options, support groups (adherence question)	5. Understanding the process through counseling, job aids, etc<br>6. Time, cost, efficiency of the standard care<br>7. Support of family, friends or support groups<br>8. Drug collection options, support groups (adherence question)

1.7 In your opinion, why do some people who should start ART not start?

Probe for:

- Personal issues: don't know, stigma, time, money
- System issues: process not easy,

Theme 3: Suggestions for improving the ART initiation process and adherence

1.8 Think about your experience with the ART initiation process. What do you suggest would improve your experience? Tell me three specific ways the process could have been easier for you? If you haven't yet initiated, imagine what might make it easy for you.

1.
2.
3.

1.9 Think about your experience with adherence to your ART medications. What do you suggest would improve your experience? Tell me three specific ways adhering could be easier for you? If you haven't yet initiated, imagine what might make it easy for you.

1.
2.
3.

1.10 We've come to the end of our interview. Is there anything else that you'd like to tell me about your experience?

Thank the participant for their time and contribution. Close the interview, turn off the recorder and complete the time the interview finished.

Time finish __________.
