# Supplementary material for: Patient Perspectives of Quality of the Same-Day Antiretroviral Therapy Initiation Process in Gauteng Province, South Africa: Qualitative Dominant Mixed-Methods Analysis of the SLATE II Trial
Source: Patient. 2020 Sep 10;14(2):175–86. doi: 10.1007/s40271-020-00437-4 (PMC7884580; doi:10.1007/s40271-020-00437-4)
Supplement: Supplementary file 2 — Supplementary material 2 (DOCX 28 kb) [file 40271_2020_437_MOESM2_ESM.docx]

**Supplement file for:**

**Patient perspectives of quality of the same-day antiretroviral therapy initiation process in Gauteng Province, South Africa: qualitative dominant mixed-methods analysis of the SLATE II trial**

Scott Nancy A^1^ ^§^, Maskew Mhairi^2^, Fong Rachel M^1^, Olson Ingrid E^1^, Brennan Alana T^1,2,3^, Fox Matthew P^1,2,3^, Vezi Lungisile^2^, Ehrenkranz Peter D^4^, Rosen Sydney B^1,2^

^1^Department of Global Health, Boston University School of Public Health, Boston, Massachusetts, United States of America

^2^Health Economics and Epidemiology Research Office, Department of Internal Medicine, School of Clinical Medicine, Faculty of Health Sciences, University of the Witwatersrand, Johannesburg, South Africa

^3^Department of Epidemiology, Boston University School of Public Health, Boston, Massachusetts, United States of America

^4^Bill & Melinda Gates Foundation, Seattle, Washington, United States of America

^§^Corresponding author:

Nancy Scott

Boston University School of Public Health: 801 Massachusetts Ave 3^rd^ Floor, Boston, MA 02118 USA, nscott@bu.edu, +1 617-358-2184

Author e-mail addresses:

NAS: [nscott@bu.edu](mailto:nscott@bu.edu)

MM: [mmaskew@heroza.org](mailto:mmaskew@heroza.org)

RMF: [rmfong@bu.edu](mailto:rmfong@bu.edu)

IEO: [iolson@bu.edu](mailto:iolson@bu.edu)

ATB: [abrennan@bu.edu](mailto:abrennan@bu.edu)

MPF: [mfox@bu.edu](mailto:mfox@bu.edu)

LV: lvezi@heroza.org

PDE: [Peter.Ehrenkranz@gatesfoundation.org](mailto:Peter.Ehrenkranz@gatesfoundation.org)

SBR: [sbrosen@bu.edu](mailto:sbrosen@bu.edu)

**Electronic Supplementary Material 1. Qualitative codebook for the qualitative dominant mixed-methods analysis of the SLATE II trial**

| Node |
| --- |
| 0. Quality of Care Framework |
| A. Elements of Quality |
| i. Choice |
| ii. Information given to clients |
| iii. Technical competence |
| iv. Interpersonal relations |
| v. Mechanisms to encourage continuity |
| vi. Appropriate constellation of services |
| vii. General procedures |
| B. Impacts |
| i. Client knowledge |
| ii. Client satisfaction |
| iii. Client health |
| iv. Service use |
| 1. Quality and Acceptability |
| A. After consent to study |
| i. General experience |
| ii. Provider interactions |
| iii. Referred |
| iv. Tests or services provided |
| B. Expectations and needs |
| i. Complicated |
| ii. Easy |
| iii. Expectations met |
| iv. More than expected |
| v. Not met |
| vi. Quality of services |
| vii. Timing |
| C. Recommend for others |
| i. A good thing |
| ii. Acceptable approach |
| iii. Not recommend |
| iv. Similar experience |
| v. Changes |
| vi. Why recommend |
| 2. Barriers and Facilitators |
| A. Facilitators |
| i. Acceptance |
| ii. Aid in understanding- counseling |
| iii. Set reminder |
| iv. Community presence |
| v. Disclosing |
| vi. Drug collection options |
| vii. Encouraged to get tested |
| viii. Family or friend support |
| ix. Feeling better |
| x. Feeling sick |
| xi. Medication |
| xii. Quality service |
| xiii. Seeing others better |
| xiv. Seeing others sick |
| xv. Self-motivation |
| xvi. Support groups |
| xvii. Work support |
| B. Barriers |
| i. Being sick day of |
| ii. Cost or food |
| iii. Drinking |
| iv. Facility service |
| v. Lack of motivation |
| vi. Lack of support |
| vii. Medication |
| viii. Not disclosing |
| ix. Not understanding or denial |
| x. Realities of life with HIV |
| xi. Stigma or fear |
| xii. Timing |
| xiii. Traditional healers |
| xiv. Travel or work |
| D. Reasons for not initiating |
| i. Personal issues |
| ii. Community issues |
| iii. Structural issues |
| 3. Suggestions for Improvement |
| A. ART initiation |
| B. ART adherence |
| C. Additional comments |
| 4. Illustrative quotes |
